# Histological findings to five years after early conversion of kidney transplant patients from cyclosporine to everolimus: an analysis from the randomized ZEUS study

**DOI:** 10.1186/s12882-018-0950-1

**Published:** 2018-06-28

**Authors:** Ute Eisenberger, Klemens Budde, Frank Lehner, Claudia Sommerer, Petra Reinke, Oliver Witzke, Rudolf P. Wüthrich, Rolf Stahl, Katharina Heller, Barbara Suwelack, Anja Mühlfeld, Ingeborg A. Hauser, Silvio Nadalin, Martina Porstner, Wolfgang Arns, on behalf of the ZEUS Study Investigators, Wolfgang Arns, Frank Lehner, Jürgen Klempnauer, Klemens Budde, Hans-H. Neumayer, Peter Gerke, Ingeborg A. Hauser, Hans Ulrich Klehr, Anja Susanne Mühlfeld, Oliver Witzke, Frank Pietruck, Katharina Heller, Petra Reinke, Norbert Senninger, Heiner H. Wolters, Barbara Suwelack, Claudia Sommerer, Martin Zeier, Rolf Stahl, Stefan Thorban, Manfred Stangl, Silvio Nadalin, Wolfgang Steurer, Ute Eisenberger, Felix Frey, Rudolf P. Wüthrich, Pierre-Alain Clavien

**Affiliations:** 1 Department of Nephrology and Hypertension, University of Bern, Inselspital, Bern, Switzerland; 2 Department of Nephrology, Charité Universitätsmedizin Berlin, Berlin, Germany; 3 Department of General, Visceral and Transplantation Surgery, Hannover Medical School, Hannover, Germany; 4 Department of Nephrology, University Hospital Heidelberg, Heidelberg, Germany; 5 Department of Nephrology and Intensive Care, Charité Campus Virchow, Charité-Universitätsmedizin Berlin, Berlin, Germany; 6 Department of Infectious Diseases, University Hospital Essen, University of Duisburg-Essen, Essen, Germany; 7 Division of Nephrology, University Hospital, Zürich, Switzerland; 8 Division of Nephrology, University Medical Center Hamburg-Eppendorf, Hamburg, Germany; 9 Department of Nephrology and Hypertension, University of Erlangen-Nuremberg, Erlangen, Germany; 10 Department of Internal Medicine - Transplant Nephrology, University Hospital of Münster, Münster, Germany; 11 Division of Nephrology and Immunology, University Hospital RWTH Aachen, Aachen, Germany; 12 Med. Klinik III, Department of Nephrology, UKF, Goethe University, Frankfurt, Germany; 13 Department of General, Visceral and Transplant Surgery, University Hospital Tübingen, Tübingen, Germany; 14 Germany Novartis Pharma GmbH, Nuernberg, Germany; 15 Department of Nephrology and Transplantation, Cologne Merheim Medical Center, Cologne, Germany; 16 Department of Nephrology, University Hospital Essen, University of Duisburg-Essen, Hufelandstr, 55, 45147 Essen, Germany

**Keywords:** Everolimus, mTOR inhibitor, Biopsy, Kidney transplantation, Randomized, Antibody-mediated rejection

## Abstract

**Background:**

Conversion from calcineurin inhibitor (CNI) therapy to everolimus within 6 months after kidney transplantation improves long-term graft function but can increase the risk of mild biopsy-proven acute cellular rejection (BPAR). We performed a *post-hoc* analysis of histological data from a randomized trial in order to further analyze histologic information obtained from indication and protocol biopsies up to 5 years after transplantation.

**Methods:**

Biopsy samples obtained up to 5 years post-transplant were analyzed from the randomized ZEUS study, in which kidney transplant patients were randomized at month 4.5 to switch to everolimus (*n* = 154) or remain on cyclosporine (CsA)-based immunosuppression (*n* = 146). All patients received mycophenolate and steroids.

**Results:**

At least one investigator-initiated biopsy was undertaken in 53 patients in each group between randomization and year 5, with a mean (SD) of 2.6 (1.7) and 2.2 (1.4) biopsies per patient in the everolimus and CsA groups, respectively. In the everolimus and CsA groups, investigator-initiated biopsies showed (i) BPAR in 12.3 and 7.5% (*p* = 0.182) of patients, respectively, with episodes graded mild in 22/24 and 18/20 cases (ii) CsA toxicity lesions in 4.5 and 10.3% of patients (*p* = 0.076) (iii) antibody-mediated rejection in 0.6 and 2.7% of patients (*p* = 0.204), respectively.

**Conclusions:**

This analysis of histological findings in the ZEUS study to 5 years after kidney transplantation shows no increase in antibody-mediated rejection under everolimus-based therapy with a lower rate of CNI-related toxicity compared to a conventional CsA-based regimen, and confirms the preponderance of mild BPAR seen in the main study after the early switch to CsA-free everolimus therapy.

**Trial registration:**

ClinicalTrials.gov NCT00154310. Date of registration: September 12, 2005.

**Electronic supplementary material:**

The online version of this article (10.1186/s12882-018-0950-1) contains supplementary material, which is available to authorized users.

## Background

Long-term graft survival is the key objective when determining the immunosuppressive regimen after kidney transplantation. The graft, however, is subject to multiple insults which can ultimately lead to irreversible nephron loss and progressive dysfunction, but this typically occurs relatively late such that graft function is a poor marker for the severity of histological changes. These include early tubulointerstitial damage from ischemia-reperfusion injury, acute and subclinical rejection, calcineurin inhibitor (CNI)-related nephrotoxicity, and viral infections [1]. Up to 1 year post-transplant, subclinical rejection is found in 30–50% of stable grafts in patients treatment with cyclosporine (CsA) [2], and in up to 15% of patients receiving tacrolimus [3–6], and is associated with subsequent tubulointerstitial damage [7, 8]. Subsequently, chronic microvascular and glomerular damage caused by interstitial fibrosis/tubular atrophy (IF/TA) becomes more common, affecting 50% of grafts by 2 years despite normal graft function [7, 8]. IF/TA is associated with reduced survival, particularly in the presence of inflammation [9].

Minimizing exposure to CNI therapy is a well-established strategy to limit pathophysiological damage to the graft [1]. Use of mammalian target of rapamycin (mTOR) inhibitors to facilitate pre-emptive CNI withdrawal after the initial post-transplant phase achieves a significant improvement in short- and long-term renal function versus a conventional CNI-based regimen if the conversion takes place by month 6 [10–13]. In two randomized trials, however, there was a significant [10] or numerical [13] increase in the rate of mild biopsy-proven acute cellular rejection (BPAR). The improved renal function during follow-up to as much as 5 years post-transplant is highly encouraging. High donor-specific antibody (DSA) values in a cohort of patients switched from CNI to everolimus have been reported in one single-center analysis [14], potentially increasing the risk for acute humoral rejection. Histological data from patients randomized to a CNI-free immunosuppressive regimen versus CNI-based long-term therapy would provide further insights into whether humoral mechanisms are relevant for pathological injury in these grafts.

Five-year follow-up data have recently been reported from the ZEUS study, in which kidney transplant patients were randomized at month 4.5 to switch to the mTOR inhibitor everolimus or remain on CsA-based immunosuppression [11]. The results showed significantly improved renal function in the everolimus-treated cohort at 5 years versus controls. BPAR occurred in 13.6 and 7.5% of patients in the everolimus and CsA cohorts, respectively (*p* = 0.095), with the difference in BPAR rates largely accounted for by grade I rejection [11]. In the ZEUS trial, graft biopsies were requested at baseline, month 12 post-transplant, and as clinically mandated. We report here a detailed analysis of the full pathology findings from the study biopsies, including the presence of CNI-related toxicity, antibody-mediated rejection (AMR) and chronic/sclerosing allograft nephropathy.

## Methods

### Study design and patient population

ZEUS was a 12-month multicenter, open-label, parallel-group trial in which kidney transplant recipients were randomized at 4.5 months post-transplant to convert to everolimus or remain on CsA therapy (NCT00154310). After completion of the 12-month study visit, patients were followed up at annual observational visits during an extension phase of the study until 5 years post-transplant. The study was undertaken at 17 transplant centers in Germany and Switzerland during June 2005 to September 2007, with the final five-year visit in September 2012. The study was conducted in compliance with Good Clinical Practice and the Declaration of Helsinki.

The study population comprised recipients of a kidney transplant aged 18–65 years. Key exclusion criteria at the time of transplantation were more than one previous renal transplantation, loss of previous graft due to immunological reasons in the first year post-transplant, multiple organ transplantation (e.g. kidney and pancreas), receipt of an organ donated after cardiac death, donor age < 5 years or > 65 years, historical or current peak panel reactive antibodies (PRA) > 25%, platelets < 75,000/mm^3^ with an absolute neutrophil count of < 1500/mm^3^ or leucocytes < 2500/mm^3^, hemoglobin < 6 g/dl, or severe liver disease. Key inclusion criteria at 4.5 months post-transplant (the point of randomization) were treatment with CsA, enteric-coated mycophenolate sodium (EC-MPS, minimum dose of 720 mg/day) and corticosteroids, and serum creatinine ≤265 μmol/l. Key exclusion criteria at month 4.5 post-transplant were graft loss, previous rejection that was severe (Banff grade ≥ II), recurrent or steroid-resistant, dialysis dependency or proteinuria > 1 g/day.

### Immunosuppression

All patients received basiliximab induction therapy (Simulect®, Novartis Pharma AG, Basel, Switzerland). Until month 4.5, the immunosuppression regimen comprised CsA (Sandimmun® Optoral/Neoral®, Novartis Pharma AG) dosed according to pre-specified target ranges for trough concentration and concentration at 2 hours post-dose [10], 1440 mg/day EC-MPS (Myfortic®, Novartis Pharma AG) and corticosteroids according to local practice.

At 4.5 months post-transplant, patients were stratified according to living or deceased donor and randomized using an automated, validated system. For patients randomized to the everolimus group, everolimus was initiated and CsA was withdrawn in a stepwise manner over a maximum of 4 weeks. The everolimus target C_0_ concentration was 6–10 ng/ml following CsA discontinuation. For patients randomized to continue CsA therapy, CsA dosing was adjusted according to tapered target ranges to month 12. After the month 12 study visit, the assigned immunosuppressive regimen was to be maintained but changes were permitted at any time based on each patient’s clinical needs at the discretion of the investigator.

### Graft biopsies

Protocol biopsies were requested at time of transplant, the point of randomization (month 4.5) and at month 12. The study protocol also stipulated that graft core biopsies were to be performed in the event of a suspected rejection episode prior to (or at the latest within 24 h after) the initiation of anti-rejection therapy. If there was an inadequate response to a full course of steroids and initiation of antilymphocyte therapy was delayed for more than 10 days after the diagnosis of BPAR, a repeat biopsy was to be performed to confirm ongoing rejection. Biopsies were read and interpreted by a local pathologist, the results of which were used without histological re-evaluation for the current analysis. Data were captured in response to the following questions: Is there any histological evidence of acute/active rejection (with Banff grading)?; Is there any histological evidence of chronic/sclerosing allograft nephropathy (with Banff grading)?; Is there any evidence of AMR (severity graded as acute tubular necrosis [ATN]-like/minimal inflammation, capillary marginal and/or thrombosis, arterial v3 [transmural inflammation/fibrinoid change])?; Is C4d staining positive?; Presence of other lesions/abnormal findings (borderline lesions, ATN, acute allograft glomerulopathy, calcineurin toxicity lesions, donor lesions, chronic allograft glomerulopathy, other)? Follow-up biopsies are reported for all patients up to 5 years post-transplant.

### Data analysis

Data were analyzed from biopsies performed at the time of randomization or thereafter in the core or extension phases of the study. Biopsies undertaken in response to a clinical event, or as follow-up to a clinical event, were categorized as ‘investigator-initiated’ and were analyzed separately from protocol-specified biopsies performed at (i) randomization or (ii) month 12 or at the point of early study discontinuation. Pathological findings according to Banff 97 criteria [15] and information on C4d staining are presented. The presence of CNI toxicity was defined according to local histopathology criteria. Histological changes characteristic of AMR are itemized. The final clinical diagnoses for all cases in which rejection was suspected and an investigator-initiated biopsy was undertaken are presented.

Data on rejection episodes were recorded during the 12-month study and at each annual follow-up visit up to 5 years post-transplant. The final clinical diagnoses of rejection events were captured up to 5 years post-transplant according to the following categories on the case report form: normal; infection; acute cellular rejection diagnosed by biopsy; acute and chronic rejection; recurrence of original disease; infarction/thrombosis; technical problem; post-transplant lymphoproliferative disorder; urinary obstruction; delayed graft function; acute tubular necrosis; urological problem; calcineurin inhibitor induced toxicity; chronic allograft nephropathy; borderline lesions; other.

The incidence of biopsy findings is presented per treatment group using two different denominators: (i) the number of patients with ≥1 biopsy (ii) the number of patients in the intent-to-treat (ITT) population. Between-group differences were compared using the T-test (two-sided), Fisher’s test (two-sided) or Chi square test where appropriate.

## Results

### Patient population and study treatment

The ITT population included 300 patients (everolimus 154, CsA 146). The 12-month core study was completed by 269 patients (138 everolimus, 131 CsA) and the five-year visit was attended by 232 patients (123 everolimus, 109 CsA) (Additional file 1: Figure S1). The characteristics of recipients and donors were similar between treatment groups other than body mass index, which was higher in the everolimus group (Additional file 1: Table S1). By year 5 post-transplant, 45.2% of patients randomized to everolimus remained on everolimus in a CNI-free regimen. In the CsA group, 58.6% of patients were still receiving CsA and a further 11 patients (7.6%) had switched to tacrolimus so remained on CNI therapy (Additional file 1: Table S2). For the patients in whom data were available regarding used of steroid therapy, 79/123 patients in the everolimus group (64.2%) and 70/109 patients in the CsA group (64.2%) were receiving steroids at 5 years post-transplant.

### Biopsy samples

Protocol biopsies were performed in 40 patients (13.3% of patients; 22 everolimus, 18 CsA) at randomization and in 34 patients (11.3% of patients; 17 everolimus, 17 CsA) at month 12. Prior to randomization at month 4.5, investigator-initiated biopsies were performed in 125 patients (41.7%), at a mean of 0.9 months post-transplant (Table 1). In total, 178 investigator-initiated biopsies were performed after randomization, in 53 everolimus-treated patients (34.4%) and 53 CsA-treated patients (36.3%), of which 67 (67/178, 37.6%) were obtained by year 1 and 111 (111/178, 62.4%) were obtained during years 2–5 (Table 1). The mean (SD) number of investigator-initiated biopsies per patient between randomization and year 5 was 2.6 (1.7) in the everolimus group compared to 2.2 (1.4) in the CsA group, at a mean of 20.2 (16.6) and 19.5 (15.8) months post-transplantation, respectively.

**Table 1:** Graft biopsies to year 5 post-transplant

	Everolimus (*n* = 154)	CsA (*n* = 146)
Number of patients with protocol-specified biopsies, n (%)		
Randomization n (%)	22 (14.3)	18 (12.3)
Month 12, n (%)	17 (11.0)	17 (11.6)
Number of patients with investigator-initiated biopsies, n (%)		
Before randomization	61 (39.6)	64 (43.8)
After randomization		
Core phase (to year 1)	27 (17.5)	26 (17.8)
Extension phase (years 2–5)	39 (25.3)	34 (23.3)
Total	53 (34.4)	53 (36.3)
Total number of investigator-initiated biopsies, n		
Before randomization	111	115
After randomization		
Core phase	33	34
Extension phase (years 2–5)	62	49
Total	95	83
Mean (SD) number of investigator-initiated biopsies per patient, n		
Before randomization	1.8 (1.0)	1.8 (1.1)
After randomization	1.8 (1.2)	1.6 (1.0)
Time from transplant to first investigator-initiated biopsy, months		
Before randomization		
Mean (SD)	0.9 (0.9)	0.9 (0.9)
Median (range)	0.4 (0.2–3.7)	0.5 (0.2–4.2)
After randomization		
Mean (SD)	19.5 (15.8)	20.2 (16.6)
Median (range)	10.5 (4.3–59.5)	13.3 (30.5–59.6)

*CsA* cyclosporine, *SD* standard deviation

An additional 54 biopsy samples (34 everolimus, 20 CsA) in 26 patients (15 everolimus, 11 CsA) were provided but were inadequately categorized regarding reason for biopsy (i.e. protocol-mandated or investigator-initiated) and were excluded from analyses. Results from these samples are shown in Additional file 1: Table S3.

### Pathology findings

#### Protocol biopsies

Protocol biopsy results at randomization and at month 12 revealed few cases of clinically undetected mild BPAR or other lesions in either treatment group, but the number of such biopsies was low (Table 2).

**Table 2:** Pathology assessment of biopsies according to Banff criteria

	No. events		% based on number of patients with ≥1 biopsy			% based on number of ITT patients		
Protocol-specified biopsies, *n* (%)	Everolimus	CsA	Everolimus	CsA	*P* value^a^			
*Biopsy at randomization*	*22*	*18*	*n = 22*	*n = 18*	*–*	*–*		
BPAR grade 1A	1	0	4.5	0	1.000	–		
Borderline lesions	4	3	18.2	16.7	1.000			
Other	1	1	4.5	5.6	1.000			
*Month 12*	*17*	*17*	*n = 17*	*n = 17*		*–*		
BPAR, any	3	1	17.6	5.9	0.601	–		
Grade 1A	0	1						
Grade 1B	1	0						
Grade 2A	2	0						
Chronic/sclerosing allograft nephropathy								
Grade 1	1	1	5.9	5.9	1.000			
CNI toxicity lesions	1	0	5.9	0	1.000			
Borderline lesions	3	1	17.6	5.9	0.601			
Investigator-initiated biopsy, *n* (%)^b^	Everolimus	CsA	Everolimus *N* = 61	CsA *N* = 64	*P* value^a^	Everolimus *N* = 154	CsA *N* = 146	*P* value^a^
*Before randomization*	*61*	*64*	*n = 61*	*n = 64*	*–*	*39.6*	*43.8*	*–*
BPAR, any	11	17	18.0	26.6	0.288	7.1	11.6	0.234
Grade 1A	9	7						
Grade 1B	0	2						
Grade 2A	1	0						
Grade 2B	0	2						
Grade 3	0	0						
Grade missing	1	7						
Antibody-mediated rejection	2	2	3.3	3.1	1.000	1.3	1.4	1.000
Chronic/sclerosing allograft nephropathy	2	2	3.3	3.1	1.000	1.3	1.4	1.000
Grade 1	1	1						
Grade 2	1	1						
Grade 3	0	0						
CNI toxicity lesions	17	20	27.9	31.3	0.700	11.0	13.7	0.489
Borderline lesions	9	15	14.8	23.4	0.260	5.8	10.3	0.202
Acute tubular necrosis	15	21	24.6	32.8	0.300	9.7	14.4	0.286
Chronic allograft glomerulopathy	0	1	0	1.6	1.000	0	0.7	0.487
Donor lesions	4	6	6.6	9.4	0.744	2.6	4.1	0.533
Other	16	21	26.2	32.8	0.440	10.4	14.4	0.380
*After randomization*	*53*	*53*	*n = 53*	*n = 53*	*–*	*34.4*	*36.3*	*–*
BPAR, any	19	11	35.8	20.8	0.131	12.3	7.5	0.182
Grade 1A	10	8						
Grade 1B	6	1						
Grade 2A	6	2						
Grade 2B	0	0						
Grade 3	1	2						
Grade missing	1	0						
Antibody-mediated rejection	1	4	1.9	7.5	0.363	0.6	2.7	0.204
Chronic/sclerosing allograft nephropathy	11	12	20.8	22.6	1.000	7.1	8.2	0.829
Grade 1	7	7						
Grade 2	2	4						
Grade 3	2	3						
CNI toxicity lesions	7	15	13.2	28.3	0.092	4.5	10.3	0.076
Borderline lesions	14	11	26.4	20.8	1.000	9.1	7.5	0.680
Acute tubular necrosis	2	1	3.8	1.9	1.000	1.3	0.7	1.000
Chronic allograft glomerulopathy	7	4	13.2	7.5	0.526	4.5	2.7	0.543
Donor lesions	0	2	0	3.8	0.495	0	1.4	0.236
Other	27	14	50.9	26.4	*0.016*	17.5	9.6	0.063

^a^Fisher’s exact test (two-sided)

^b^More than one biopsy was performed in some patients, so more than one grade per patient could be specified

*BPAR* biopsy-proven acute cellular rejection, *CNI* calcineurin inhibitor, *CsA* cyclosporine, *ITT* intent-to-treat

#### Investigator-initiated biopsies prior to randomization

In the 125 patients who underwent biopsy prior to randomization, BPAR was present in 28 cases (22.4%), the majority of which were grade 1A (Table 2). CNI-related lesions (*n* = 37) and acute tubular necrosis (*n* = 36) were more frequently observed than BPAR. AMR was rare (4/125, 3.2%).

#### Investigator-initiated biopsies after randomization

Among patients in whom post-randomization investigator-initiated biopsies were performed between randomization and year 5, BPAR was detected in 19 everolimus-treated patients (19/154, 12.3%; 10 episodes of grade 1A, 6 grade 1B, 6 grade 2A, 1 grade 3 [1 missing]) and 11 CsA-treated patients (11/146, 7.5%; 8 episodes of grade 1A, 1 grade 1B, 2 grade 2A, 2 grade 3) (*p* = 0.182) (Table 2). The incidence from randomization to year 1 was 8.4% (13/154) versus 3.4% (5/146) in everolimus and CsA groups, respectively (*p* = 0.088), and 5.8% (9/154) versus 4.8% (7/146) during the extension phase (years 2–5) (*p* = 0.799). The severity of BPAR between randomization and year 5 was comparable between groups, with 22/24 episodes of BPAR in the everolimus group and 18/20 episodes in the CsA group graded mild (grade ≤ 2A).

The proportion of patients exhibiting chronic/sclerosing allograft nephropathy on investigator-initiated biopsies after randomization was 7.1% in the everolimus group and 8.2% in the CsA group (*p* = 0.829), and was graded mild (Banff grade ≤ 2A) in the majority of cases (Table 2). Borderline lesions occurred with similar frequency in each group (*p* = 0.680). Pathologists reported CNI toxicity lesions in 7/154 (4.5%) and 15/146 (10.3%) of patients undergoing investigator-initiated biopsies in the everolimus and CsA groups, respectively (*p* = 0.076).

Post-randomization, AMR was detected in one everolimus-treated patient (0.6%) which was categorized as ATN-like and was C4d positive. Four CsA-treated patients developed AMR (2.7%): two showed ATN-like changes (both C4d positive), one had arterial-v3 changes (C4d positive) and one showed capillary-marginal change/thrombosis (C4d negative). C4d staining was detected in 17 everolimus-treated patients (11.0%) and 10 CsA-treated patients (6.8%), including one of the three patients in the everolimus group who developed AMR and three of the six CsA-treated patients with AMR.

When pathology diagnoses in investigator-initiated biopsy samples after randomization were compared as a proportion of patients undergoing biopsy, there was a higher rate of ‘other’ lesions in the everolimus cohort (50.9% versus 26.4%, *p* = 0.016) and a non-significant trend (*p* = 0.092) to a higher rate of CNI-related toxicity lesions in the CsA arm.

### Clinical diagnoses following biopsy

The final clinical diagnosis among patients with one or more investigator-initiated biopsy after randomization performed in response to suspected rejection (everolimus 52, CsA 50) is summarized in Table 3.

**Table 3:** Final clinical diagnosis following a post-randomization investigator-initiated biopsy in response to suspected rejection^a^

	No. events		% based on number of patients with ≥1 suspected rejection			% based on number of ITT patients		
	Everolimus	CsA	Everolimus *N* = 52	CsA *N* = 50	*P* value^b^	Everolimus *N* = 154	CsA *N* = 146	*P* value^b^
Acute cellular rejection^c^	17	26	32.7	52.0	0.071	11.0	17.8	0.102
Acute & chronic rejection	1	2	1.9	4.0	0.614	0.6	1.4	0.614
Borderline lesions	17	17	32.7	34.0	1.000	11.0	11.6	1.000
CNI-induced toxicity	14	14	26.9	28.0	1.000	9.1	9.6	1.000
Acute tubular necrosis	4	6	7.7	12.0	0.521	2.6	4.1	0.533
Chronic allograft nephropathy	9	9	17.3	18.0	1.000	5.8	6.2	1.000
Infection	0	3	0	6.0	0.114	0	2.1	0.114
Normal	13	10	25.0	20.0	0.638	8.4	6.8	0.668
Other	23	20	44.2	40.0	0.692	14.9	13.7	0.869

^a^More than one clinical diagnosis was possible

^b^Fisher's exact test (two-sided)

^c^Includes diagnosis made in advance of biopsy results (everolimus 2, CsA 1)

*CNI* calcineurin inhibitor, *CsA* cyclosporine

## Discussion

Clinically-indicated renal biopsies from the randomized ZEUS study up to year 5 post-transplant showed a trend to more frequent mild acute cellular rejection, and approximately half the incidence of CNI-toxicity lesions, under an everolimus-based regimen with early CNI elimination versus standard CsA therapy. The between-group differences were not statistically significant, however, in the relatively small cohort of patients undergoing clinically-indicated biopsy after randomization. The severity of acute cellular rejection and rates of AMR and chronic allograft nephropathy were similar between groups following randomization. Consistent with this, the proportion of patients in whom a post-randomization biopsy was requested was also comparable with everolimus- or CsA-based treatment.

The surveillance biopsies which were requested in the study protocol at randomization and month 12 were performed in only a low proportion of patients, severely limiting interpretation. There was a numerically higher number of BPAR and borderline lesions in the everolimus group, but these were each observed in only three patients versus one patient in the CsA group, so conclusions cannot be drawn.

AMR was rare in both treatment arms after randomization (everolimus 0.6%, CsA 2.7%). The diagnosis was made based only on histological changes with or without C4d staining [15], since, at that time, DSAs were not measured in most centers during the study. A subsequent protocol amendment specified that data on DSA levels should be collected at the five-year study visit, but this was provided for only 28 patients in the everolimus group and 25 patients in the CsA group, and, in this subset of patients, no difference was found between treatment groups [11]. In the recent CENTRAL study, which randomized kidney transplant patients at week 7 to switch to everolimus or remain on CsA, DSA was detected in 15.0% of patients in the everolimus group (9/60 patients) and 21.1% in the CsA arm (12/57 patients) (*p* = 0.600) [16].

The incidence of CNI-induced toxicity based on histological analysis was approximately twice as high in the CsA cohort as in those switched to everolimus, but the absolute difference was relatively small (4.5% versus 10.3%). This is perhaps not unexpected since it is accounted for by the fact that the first investigator-initiated biopsy was performed at approximately 20 months post-transplant in both groups. The histological lesions which characterize CNI nephrotoxicity (arteriolar hyalinosis, striped cortical fibrosis, tubular microcalcification) develop progressively over time [17]. In a study of sequential protocol biopsies, Nankivell et al. showed that approximately 25% of patients had CNI-induced lesions by month 6, doubling to half of all patients by year 5 [17], but in our series the low rate of protocol biopsies was inadequate to offer meaningful data on subclinical CNI-related nephrotoxicity.

Protocol-specified biopsies were requested but were not mandatory, and were performed in accordance with local center practice. In fact, protocol biopsies were performed in only 13% of patients at randomization and in only 11% at month 12, presumably since this conflicted with local practice. Regrettably, biopsy data from 26 patients did not specify whether they were protocol- or clinically-mandated, and were therefore excluded from the current analysis since they could not reliably be assigned to a category. The resulting small numbers preclude reliable interpretation and negate the possibility for a matched/paired analysis. Previously, the CERTITEM study has reported a higher rate of subclinical rejection in kidney transplant patients switched from CNI therapy to everolimus at month 3 post-transplant versus those who continued CNI (10.4% versus 2.0%, *p* = 0.015) [18]. In that trial, however, the everolimus-treated population was under-immunosuppressed due to 50% reduction in mycophenolic acid dose. Here, a difference in subclinical rejection appears unlikely since there was no indication of greater long-term histological deterioration under everolimus based on investigator-initiated biopsies and since renal function remained superior to the CNI treatment group to 5 years post-transplant [11].

The ZEUS trial offered the benefit of a randomized, multicenter study design with long-term follow-up to 5 years, and a balanced proportion of patients in each treatment group providing at least one biopsy sample after randomization. All pathological assessments were carried out locally at the 17 participating centers to ensure rapid information for clinical decision-making, and inevitably this introduces the risk of variability in pathological assessments between centers [19, 20]. There is no reason to expect, however, that this variability influenced the between-group comparison. Also, more than 60% of investigator-initiated biopsies were undertaken after year 1, offering a good comparison of the effect of the two treatment regimens on graft histology over the first 5 years after kidney transplantation. It is also important to note that by the end of the five-year follow-up period, only 45% of patients in the everolimus arm were still receiving everolimus, and only 59% of those randomized to CsA continued to receive CsA.

Clinical analyses of data from the ZEUS study at one [10] and five [11] years post-transplant have shown superior graft function after switch from CsA to everolimus therapy, with a higher rate of mild acute cellular rejection reported by clinicians in the everolimus cohort at year 1. This more detailed analysis of histological findings from the ZEUS study confirms the preponderance of early mild BPAR cases in the CNI-free everolimus-arm as published before [10], but with no increase in AMR and a lower rate of CNI-related toxicity. These findings suggest that conversion from CsA to everolimus by month 6 after kidney transplantation, with concomitant mycophenolic acid and steroids, can be undertaken safely and offers the possibility to reduce CNI-related toxicity.

## Additional file

Additional file 1: **Table S1.** Baseline characteristics (safety population). **Table S2.** Immunosuppression at 5 years post-transplant (safety population), *n* (%). **Table S3.** Pathology assessment of biopsies according to Banff criteria in patients with ≥1 biopsy not categorized as ‘protocol-specified’ or ‘investigator-initiated’. **Figire S1.** CsA, cyclosporine. (DOCX 129 kb)

## Abbreviations

AMR: Antibody-mediated rejection
ATN: Acute tubular necrosis
BPAR: Biopsy-proven acute cellular rejection
CNI: Calcineurin inhibitor
CsA: Cyclosporine
DSA: Donor-specific antibody
EC-MPS: Enteric-coated mycophenolate sodium
IF/TA: Interstitial fibrosis/tubular atrophy
ITT: Intent-to-treat
mTOR: Mammalian target of rapamycin
