# Supplementary material for: Histological findings to five years after early conversion of kidney transplant patients from cyclosporine to everolimus: an analysis from the randomized ZEUS study
Source: BMC Nephrol. 2018 Jun 28;19:154. doi: 10.1186/s12882-018-0950-1 (PMC6025714; doi:10.1186/s12882-018-0950-1)
Supplement: Supplementary file 1 — Table S1. Baseline characteristics (safety population). Table S2. Immunosuppression at 5 years post-transplant (safety population), n (%). Table S3. Pathology assessment of biopsies according to Banff criteria in patients with ≥1 biopsy not categorized as ‘protocol-specified’ or ‘investigator-initiated’. Figire S1. CsA, cyclosporine. (DOCX 129 kb) [file 12882_2018_950_MOESM1_ESM.docx]

**Histological findings to five years after early conversion of kidney transplant patients from cyclosporine to everolimus: An analysis from the randomized ZEUS study**

Eisenberger U et al

**Additional files**

**Additional Table S1**. Baseline characteristics (safety population)

|  | **Everolimus (n=155)^a^** | **CsA (n=145)^a^** | **P value^b^** |
| --- | --- | --- | --- |
| Age (years) | 46.9 (11.7) | 46.7 (11.9) | 0.883 |
| Male gender, n (%) | 102 (66) | 86 (59) | 0.282 |
| Ethnic origin, n (%)  White  Asian  Other | 152 (98)  2 (1)  1 (<1) | 139 (96)  4 (3)  2 (1) | 0.323 |
| Body mass index (kg/m^2^) | 25.6 (4.0) | 24.4 (4.0) | **0.010** |
| End-stage disease leading to transplantation, n (%)   Glomerulonephritis/glomerular disease  Polycystic disease  IgA nephropathy  Hypertension or nephrosclerosis  Pyelonephritis  Other  Unknown | 42 (27)  27 (17)  19 (12)  11 (7)  11 (7)  39 (25)  6 (4) | 47 (32)  24 (17)  17 (12)  9 (6)  4 (3)  38 (26)  6 (4) | 0.715 |
| Pretransplant dialysis, n (%) | 141 (91) | 137 (94) | 0.274 |
| Number of previous renal transplants, n (%)  0  1  2 | 134 (86)  21 (14)  0 | 122 (84)  19 (13)  4 (3) | 0.626 |
| Panel-reactive antibodies >5% at most recent assessment, n (%) | 1 (<1) | 4 (3) | 0.201 |
| Cold ischemia time (h) (deceased donors) | 14.0 (5.7) | 14.6 (5.0) | 0.409 |
| Diabetes at baseline, n (%) | 13 (8) | 12 (8) | 1.000 |
| Donor age (years) | 47.5 (12.4) | 47.6 (13.1) | 0.946 |
| Donor type, n (%)  Living related  Living unrelated  Deceased | 27 (17)  15 (10)  113 (73) | 30 (21)  8 (6)  107 (74) | 0.346 |

Continuous variables are shown as mean (SD)

^a^ One patient randomized to CsA was given everolimus in error
^b^ T-test (two-sided), Fisher's test (two-sided) or Chi square test as appropriate

CsA cyclosporine; SD, standard deviation

**Additional Table S2**. Immunosuppression at five years post-transplant (safety population), n (%)

|  | **Everolimus (n=155)^a^** | **CsA (n=145)^a^** |
| --- | --- | --- |
| Everolimus | 70 (45.2) | 10 (6.9) |
| CsA | 19 (12.3) | 85 (58.6) |
| Tacrolimus | 28 (18.1) | 11 (7.6) |
| Everolimus + tacrolimus | 1 (0.6) | 1 (0.7) |
| Sirolimus | 1 (0.6) | 4 (2.8) |
| EC-MPS monotherapy | 2 (1.3) | 0 |
| Missing data | 34 (21.9) | 34 (23.4) |

^a^ One patient assigned to CsA was given everolimus in error

CsA, cyclosporine; EC-MPS, enteric-coated mycophenolate sodium

**Additional Table S3.** Pathology assessment of biopsies according to Banff criteria in patients with ≥1 biopsy not categorized as 'protocol-specified' or 'investigator-initiated'.

|  | **n (%)** | |
| --- | --- | --- |
|  | **Everolimus**  **(N=15)** | **CsA (N=11)** |
| BPAR, any   Grade 1A  Grade 1B | 0 2 | 1 0 |
| Antibody-mediated rejection | 0 | 0 |
| Chronic/sclerosing allograft nephropathy   Grade 1   Grade 2  Grade 3 | 2 0 0 | 0 1 0 |
| CNI toxicity lesions  Borderline lesions  Acute tubular necrosis  Donor lesions  Other | 3 8 1 0 1 | 3 3 2 1 3 |

**Additional Figure S1**CsA, cyclosporine
